# The Shape Trail Test: Application of a New Variant of the Trail Making Test

**DOI:** 10.1371/journal.pone.0057333

**Published:** 2013-02-20

**Authors:** Qianhua Zhao, Qihao Guo, Fang Li, Yan Zhou, Bei Wang, Zhen Hong

**Affiliations:** 1 Department of Neurology, Huashan Hospital, Fudan University, Shanghai, China; 2 Department of Gerontology, Fuxing Hospital, Capital Medical University, Beijing, China; Banner Alzheimer's Institute, United States of America

## Abstract

**Objective:**

The Trail making test (TMT) is culture-loaded because of reliance on the Latin alphabet, limiting its application in Eastern populations. The Shape Trail Test (STT) has been developed as a new variant. This study is to examine the applicability of the STT in a senile Chinese population and to evaluate its potential advantages and disadvantages.

**Method:**

A total of 2470 participants were recruited, including 1151 cognitively normal control (NC), 898 amnestic mild cognitive impairment (aMCI), and 421 mild Alzheimer disease (AD) patients. Besides the STT, the Mini mental state examination and a comprehensive neuropsychological battery involving memory, language, attention, executive function and visuospatial ability were administered to all the participants. In a subgroup of 100 NC and 50 AD patients, both the STT and the Color Trail Test (CTT) were performed.

**Results:**

In NC, the time consumed for Part A and B (STT-A and STT-B) significantly correlated with age and negatively correlated with education (p<0.01). STT-A and B significantly differed among the AD, aMCI and NC. The number that successfully connected within one minute in Part B (STT-B-1 min) correlated well with STT-B (r = 0.71, p<0.01) and distinguished well among NC, aMCI and AD. In the receiver operating characteristic curve analysis, the AUCs (area under the curve) for STT-A, STT-B, and STT-B-1min in identifying AD were 0.698, 0.694 and 0.709, respectively. The STT correlated with the CTT, but the time for completion was longer.

**Conclusion:**

The TMT is a sensitive test of visual search and sequencing. The STT is a meaningful attempt to develop a “culture-fair” variant of the TMT in addition to the CTT.

## Introduction

The Trail Making Test (TMT) was originally constructed in 1938 as “Partington's Pathway” or the “Divided Attention Test” 1]. Scoring is expressed in terms of the time in seconds for Part A and Part B of the test. Some examiners also calculate a Trails B/Trails A ratio. The TMT is one of the most sensitive and popular tests for identifying mild cognitive impairment and mild dementia 2,3,4,5,6]. It measures the speed for attention, sequencing, mental flexibility, and of visual search and motor function, mainly reflecting the ability of “set shifting” 7,8,9,10]. Set shifting is a main component of executive function, involving the control of endogenous attention, which refers to the cognitive strategy of individuals to shift the focus of their attention between various external stimuli in the face of two cognitive tasks competing for same mental resources 11].

**Figure 1:**
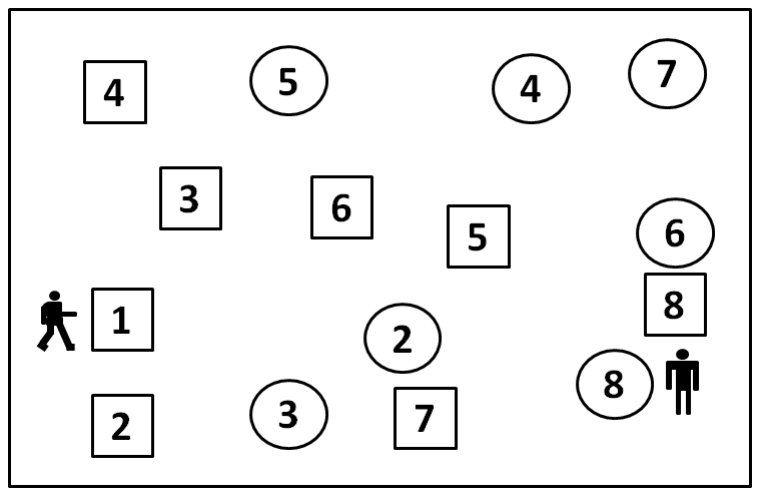
Shape Trail Test: Part B-Practice Test.

The original TMT is a frequently administered neuropsychological test in English-speaking countries with limited utility in cross-culture contexts because of the use of the English alphabet on Trail B12]. It requires the connection, by making pencil lines, between 25 enriched numbers randomly arranged on a page, in proper order (Part A) and of 25 enriched numbers and English letters in alternating order (Part B) (1-A-2-B-). Many Chinese elderly, especially the poorly-educated elderly, cannot read English letters, resulting in a very low accomplishment ratio with the classic TMT. In China, the Shape Trail Test (STT) 13] and the Color Trail Test (CTT) 14] are more widely used. The CTT and STT were developed with the intent to minimize cultural bias and provide a more accurate cognitive measure in diverse populations. The CTT requires the subjects to connect numbers alternating between two different colors (pink and yellow), while in the STT, Part B shows all numbers (from 1 to 25) twice, once in a circle and once in a square. The participants are asked to make lines alternating between circles and squares and disregarding the numbers of the alternate shapes.(See Figure 1) The reliability and validity of the CTT have been established 15,16,17,18], while for the STT, such data are still absent.

The aim of this study is to examine the applicability of STT in Chinese senile population, evaluate its potential advantages and disadvantages, and provide evidence for future research.

## Materials and Methods

### Participants

A total of 2470 participants were recruited, including 1151 cognitively normal controls (NC), 898 patients with amnestic mild cognitive impairment (aMCI), and 421 patients with mild Alzheimer disease (AD).

We recruited the controls using cluster sampling in Jingansi Community Shanghai, China. The inclusion criteria for NC were: age between 50 and 90; no memory complaints verified by an informant; cognitively normal, based on the absence of significant impairment in cognitive functions or activities of daily living (ADL); Clinical Dementia Rating (CDR)  =  0; and Hamilton depression rating scale (HAMD) scored ≤ 12 on the 17-item scale in past 2 weeks. They had adequate visual and auditory acuity to allow cognitive testing. Participants with any significant neurologic disease and psychiatric disorders/psychotic features were excluded.

All the patients with aMCI and AD were recruited from the Memory Clinic, Huashan Hospital, from Jun 2004 to Oct 2011.They finished the laboratory tests and cranial CT/MRI scan, and had no clinically significant abnormalities in vitamin B12, folic acid, thyroid function (free triiodothyronine-FT3, free tetraiodothyronine-FT4, thyroid stimulating hormone-TSH), rapid plasma regain (RPR), or treponema pallidum particle agglutination (TPPA).

The aMCI patients were diagnosed according to the following criteria19]: (1) cognitive complaints verified by an informant; (2) cognitive impairment lasting more than 3 months; (3) Mini-mental state examination-Chinese version (C-MMSE)[25] ≥ cut-off score for adjusted education; (4) Abnormal objective memory impairment documented by scoring below the age and education adjusted cutoff on an episodic memory test (Auditory Verbal Learning Test); (5) preserved basic ADL/minimal impairment in complex instrumental functions; (6) etiology unknown; (7) normal sense of hearing and sight; (8) has not met diagnostic criteria of dementia based on the National Institute of Neurological and Communicative Disorders and Stroke and the Alzheimer's Disease and Related Disorders Association (NINCDS-ADRDA).

The AD patients (n = 421) met the following criteria: (1) diagnosed as probable AD according to the NINCDS-ADRDA; (2) no obvious medical, neurological or psychiatric diseases or psychological dysfunction including anxiety and depression within the previous one month; (3) no visual or auditory deficit.

### Ethical issues

The study was approved by the Independent Reviewing Board (IRB) of Huashan Hospital. Each subject or the proxy of the AD patient signed informed consent.

### Measures

Participants were given neuropsychological tests by a trained rater who was blind to diagnosis. Besides MMSE, a comprehensive neuropsychological battery involving memory, language, attention, executive function and visuospatial ability was used. The tests were as follows: the Auditory Verbal Learning Test (AVLT), the Rey-Osterrieth Complex Figure Test (CFT), the Boston Naming Test (BNT; the 30-item version), the Animal verbal fluency test (AFT), the Symbol Digit Modalities Test(SDMT), the STT (including Part A and B), the Stroop Color-Word Test (SCWT), the Similarity test, the Clock-drawing test (CDT), the CDR and HAMD. All tests have been proven to have a good reliability and validity in Chinese.

### Statistical analysis

Chi-square analysis was adopted for ordinal data. Overall differences among the groups were assessed with one-way analysis of variance (ANOVA). Post hoc pairwise comparisons between groups were assessed using the LSD test. The level of significance was set at α = 0.05. Pearson correlation was used to evaluate the relationship analysis. The receiver operating curve was used to assess the sensitivity, specificity and cut-off score. The area under the ROC curves (AUC) was used as an overall index of performance of the screening tests. The AUCs and their standard errors were calculated using the method of Hanley and McNeil.

## Results

### 1. Basic characteristics of the STT

In the total of 2470 subjects, 188 (7.6%) could not complete the STT-Part B. Specifically, in the NC, aMCI and AD groups, 17 (1.5%), 43 (4.8%), and 128 (30.4%) failed in Part B. As such, a “floor effect” of the STT-Part B was observed in AD patients.

We analyzed the correlation between the STT and demographic variables in NC subjects. The time consumed for Part A and B (STT-A, STT-B) correlated with age (r = 0.204, 0.300) (p<0.01) and negatively correlated with education (r = −0.208, −0.161) (p<0.01). We then analyzed the gender difference in four subgroups (Group 1: age< = 65yrs, formal education year< = 12yrs; Group 2: age< = 65yrs, education>12yrs; Group 3: age>65yrs, education< = 12yrs; Group 4: age>65yrs, education>12yrs.). In younger participants with lower education (Group 1), men perform better than women statistically in both STT-A and STT-B (STT-A, M: 49.76±14.40, F: 60.21±23.23, p = 0.000; STT-B, M: 131.86±38.50, F: 162.82±51.59, p = 0.000). In older NC participants with higher education (Group 4), women need more time to complete STT Part B than men (STT-B, M: 150.01±45.88, F: 167.29±52.88, p = 0.003). In other subgroups, we didn't find any statistically significant gender difference.

In the NC group, the STT correlated well with other neuropsychological tests (p<0.01). The STT-A correlated more closely than the STT-B with the BNT, AFT, and DSST, which reflected language and attention. Meanwhile, the STT-B correlated more closely than the STT-A with the AVLT and SCWT, which reflected memory and executive function. The STT-A correlated well with the STT-B (r = 0.543, p<0.01). Both indexes correlated with the MMSE (r = 0.521, 0.426) (p<0.01).

### 2. Comparison among NC, aMCI and mild AD stratified by age and education

The performances of the STT Parts A and B were influenced by age and education. Thus, in the following analysis, participants were stratified as following: Group 1: age< = 65yrs, formal education year< = 12yrs; Group 2: age< = 65yrs, education>12yrs; Group 3: age>65yrs, education< = 12yrs; Group 4: age>65yrs, education>12yrs. The STT-A and B were significantly different among the three diagnostic groups (AD>aMCI>NC). (Results see Table 1).

**Table 1:** Comparison among NC, aMCI and mild AD stratified by age and education.

	Group 1				Group 2				Group 3				Group 4			
	NC	aMCI	AD	F(P)	NC	aMCI	AD	F(P)	NC	aMCI	AD	F(P)	NC	aMCI	AD	F(P)
N	336	206	86		313	192	72		201	236	138		301	264	125	
Age	58.0 (4.4)	57.3 (4.8)	56.8 (5.1)	2.75 (0.06)	60.7 (5.0)	60. 4 (5.4)	59.3 (4.7)	2.16 (0.12)	74.3 (4.1)	74.8 (4.1)	75.2 (4.5)	2.11 (0.12)	73.5 (5.5)	73.9 (4.6)	74.6 (4.1)	2.25 (0.10)
Education	8.7 (1.9)	9.0 (1.6)	8.7 (1.8)	2.28 (0.10)	13.7 (1.6)	13.6 (1.7)	13.2 (1.4)	2.23 (0.11)	9.5 (2.0)	9.6 (2.4)	9.1 (2.6)	2.28 (0.10)	14.2 (1.8)	14.4 (1.8)	14.0 (1.6)	2.65 (0.07)
Sex	173:163	89:117	41:45	1.76 (0.17)	167:146	108:84	33:39	1.14 (0.32)	99:102	129:107	72:66	0.63 (0.53)	172:129	164:100	72:53	0.79 (0.45)
MMSE	28.1 (1.7)##	27.0 (1.9)**	21.0 (3.1) ††	421.9 1 (0.00)	28.5 (2.8)##	27.2 (1.9)**	21.2 (3.2) ††	220.62 (0.00)	27.5 (1.7)##	26.4 (2.0)**	20.8 (2.6) ††	445.50 (0.00)	28.0 (1.7)##	26.8 (1.7)**	20.8 (2.9) ††	580.8 (0.00)
STT-A	54.8 (19.8)##	70.0 (32.3)**	132.4 (75.1) ††	155.85 (0.00)	49.3 (17.4)##	70.6 (33.6)**	117.7 (73.1) ††	117.40 (0.00)	65.2 (24.5)##	88.3 (40.9)**	119.3 (59.0) ††	69.04 (0.00)	55.8 (17.9)##	74.1 (32.9)**	120.0 (69.1) ††	128.4 (0.00)
STT-B	146.8 (47.8)##	198.6 (103.3)**	289.1 (118.6) ††	84.72 (0.00)	136.3 (50.2)##	195.3 (84.3)**	292.0 (133.1) ††	112.22 (0.00)	197.3 (62.6)##	259.0 (95.8)**	321.8 (134.1) ††	59.81 (0.00)	157.5 (49.7)##	219.2 (91.9)**	285.0 (123.2) ††	98.60 (0.00)

Group 1: age ≤65yrs, formal education years ≤12yrs; Group 2: age ≤65yrs, education >12yrs; Group 3: age >65yrs, education ≤12yrs; Group 4: age >65yrs, education >12yrs.

The data are presented as mean (Standard Deviation).

Comparison between NC group and aMCI group was marked behind “aMCI group”,

** P<0.01.

Comparison between aMCI group and AD group was marked behind “AD group”,

†† P<0.01.

Comparison between NC group and AD group was marked behind “NC group”,

## P<0.01.

### 3. ROC analysis for the STT in identifying AD among NC

We established the cut-off score according to age and education. As we can see from Table 2, in Group 1 and Group 4, which were the younger subjects with lower education or the older population with higher education, the cut-off score for the STT-A was 80 seconds. For those younger subjects with higher education, the cut-off score decreased to 70 seconds whereas for the older participants with lower education, the cut-off score increased to 90 seconds. Similarly, with regard to the STT-B, the cut-off score for Group 1 and 4 was 220 seconds; for group 2 it was 200 seconds while for Group 3 it was 240 seconds. (see Table 2).

**Table 2:** Receiver Operating Characteristic Curve Analysis for STT for Identifying AD.

		AUC&	95% CI	Cut-off score(s)^<$>\scale50%\raster="rg1"<$><$>\scale50%\raster="rg1"<$>^	Sensitivity (%)	Specificity (%)	Z (P)#
Group 1	STT-A^$^	0.830	0.896–0.765	>80	91.7	72.1	0.36(0.71)
	STT-B^%^	0.913	0.959–0.866	>220	92.4	75.0	
Group 2	STT-A	0.906	0.947–0.864	>70	87.2	77.8	1.38(0.16)
	STT-B	0.880	0.939–0.821	>200	90.7	72.5	
Group 3	STT-A	0.835	0.878–0.791	>90	84.6	66.7	1.38(0.16)
	STT-B	0.816	0.870–0.762	>240	76.4	69.9	
Group 4	STT-A	0.877	0.916–0.838	>80	88.4	66.4	1.07(0.28)
	STT-B	0.874	0.915–0.832	>220	89.5	67.0	

Group 1: age ≤65yrs, formal education years ≤12yrs; Group 2: age ≤65yrs, education >12yrs; Group 3: age >65yrs, education ≤12yrs; Group 4: age >65yrs, education >12yrs.

& AUC: area under the curve; ^$^STT-A: time consumed for STT-Part A; ^%^STT-B: time consumed for STT-Part B; ^
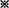
^cut-off score in seconds.

# Comparison between STT-A and B of AUC.

### 4. One minute index analysis

The numbers that the participants connected in the STT-Part B within the first one minute (STT-B-1 min) were also analyzed in a sub-sample of this study. A total of 474 (NC: 172; aMCI: 241; AD: 61) subjects finished this part of analysis.

The STT-B-1 min correlated well with the STT-B (r = 0.71, p<0.01) and distinguished well among NC, aMCI and AD subjects (Table 3). In the ROC analysis, the AUCs for the STT-A, STT-B, STT-B-1min were 0.698, 0.694 and 0.709, respectively, for identifying AD. There was no significant difference between each of the two indexes (e.g., the STT-B vs STT-B-1min, z = 0.89, p = 0.37). In 196 participants who could not complete the STT-Part B, the STT-B scores were missing, but 5 of these participants still got an STT-B-1min score.

**Table 3:** STT-B-1 min in Three Different Diagnostic Groups.

	NC	aMCI	AD	F(P)
N	172	241	61	
MMSE	28.8(3.5) ^##^	27.2(1.8)^**^	22.1(2.5) ^††^	145.7(0.00)
STT-A	52.1 (17.4) ^##^	68.7 (27.6)^**^	90.09 (43.0) ^††^	47.46(0.00)
STT-B	151.9 (62.0)^##^	201.5 (84.5)^**^	266.8(112.1)^††^	46.60(0.00)
STT-B-1min	11.2 (4.0)^##^	8.2 (3.8)^**^	6.49 (3.2) ^††^	47.24(0.00)

Comparison between NC group and aMCI group was marked behind “aMCI group”, ^**^ P<0.01.

Comparison between aMCI group and AD group was marked behind “AD group”, ^††^ P<0.01.

Comparison between NC group and AD group was marked behind “NC group”, ^##^ P<0.01.

### 5. Comparison between the STT and the CTT

One hundred elderly NC (n = 100) and fifty mild AD (n = 50) patients completed both the STT and CTT. There were no significant differences in age, education and gender between the two groups (age: 65.9±8.6 vs 68.1±7.6; edu: 12.1±3.1 vs 11.8±2.9; gender M/F: 45/55 vs 24/26, p>0.05). The MMSE scores for them were 28.0±1.5 and 24.7±2.8, respectively (t = 7.72, P<0.01). The STT and CTT correlated well with each other.(See Table 4) But the time consumed for the STT Part A was shorter than the CTT Part A, while those for the STT Part B were longer than those for the CTT Part B, indicating that the interference burden in the STT Part B (which is shape) was heavier than that in the CTT Part B (which is color).(See Table 5)

**Table 4:** Correlation between Shape Trail Test and Color Trail Test.

Correlation	STT-A	STT-B	CTT-A	CTT-B
STT-A	1	0.522**	0.502**	0.526**
STT-B		1	0.512**	0.565**
CTT-A			1	0.546**
CTT-B				1

STT-A: time consumed for the Shape Trail Test (STT)-Part A; STT-B: time consumed for the STT-Part B; CTT-A: time consumed for the Color Trail Test (CTT)-Part A; CTT-B: time consumed for the CTT-Part B.

** p<0.01.

**Table 5:** Performance of Shape Trail Test and Color Trail Test between NC and mild AD.

	NC(n = 100)	Mild AD (n = 50)	t	P
STT-A	63.2±24.0	76.2±34.9	2.37	0.020
STT-B	182.6±71.0	230.5±83.5	3.44	0.001
CTT-A	78.0±47.8	95.5±47.4	2.12	0.036
CTT-B	138.4±44.2	190.3±65.2	5.03	0.000

STT-A: time consumed for the Shape Trail Test (STT)-Part A; STT-B: time consumed for the STT-Part B; CTT-A: time consumed for the Color Trail Test (CTT)-Part A; CTT-B: time consumed for the CTT-Part B.

## Discussion

Although the TMT is one of the most widely used measures in neuropsychological practice, it is culture-loaded because of reliance on the Latin alphabet, which has limited its application in Eastern populations 20]. The recent variant form of the TMT is the CTT, which is designed to minimize the influence of language and covers the full children-to-adult age range. It has allowed broader application for cross-cultural studies, while at the same time being similar to the original TMT in terms of neuropsychological sensitivity. However, studies have also suggested that there might be a large variability in performance on the CTT because of culture-bound factors. Familiarity with testing procedures and relevance of the applied techniques to real-life experience could affect task performance 15,18]. Besides, the possibility of a Stroop effect on CTT-B performance, as suggested by Spreen and Strauss1], may influence test performance in a way that makes the CTT-Part B a qualitatively different task in comparison to the classic TMT-Part B. Additionally, the CTT requires four color-printed record sheets which increases the cost accordingly.

The STT is another variant form of the original TMT. It was developed by Agnes Chan from the Chinese University of Hong Kong13]. In the current study, we analyzed the correlation between the STT with demographic variables in cognitively normal individuals, which showed a similar trend to the TMT 3,21,22,23,24,25]. The test loaded on both “rapid visual search” and “visuospatial sequencing” factors, as well as “cognitive set-shifting”. Factor analysis revealed that the STT-A and B correlated only moderately (r = 0.522) with each other, suggesting that they measure somewhat different functions, which is similar to the original TMT. In addition to switching between squares and circles, Part B also includes more visual interferences and a longer path length. Hence, Part B requires more visual perceptual processing ability than Part A. Our factor analysis revealed that the STT-A reflected language and attention while the STT-B relied more on executive function and memory.

The trail test has been reported to be sensitive to neurological disorders such as closed-head injury, alcoholism and substance abuse. Studies have also found significant differences among AD, MCI and controls in the TMT26]. Similarly, in the current study, the STT-A and B distinguished well among AD, aMCI and controls. When age and education-stratified cut-off scores were adopted, the AUC of the ROC curves of the STT-A and B ranged from 0.816–0.913, and the sensitivity and specificity were also acceptable. We did not find any significant difference between the STT-A and B in differential validity.

The scoring of the original TMT has several indexes: (1) time in seconds required to complete each part1]; (2) derived indices from completion time: difference score  =  B-A, ratio score = B/A, Log ratio score =  Log B: A, and proportional score  =  (B-A)/A 27]; (3) Errors: the number of wrong connections, including number of errors, near misses and corrections 26]; and (4) Prompts: the number of reminding times during connection 1]. Another scoring method sets a time limit on part B of 4 minutes. The respondent is scored 25 if he completes all the connections within 4 minutes. If not, his score is the number that he finished connecting 28,29]. However, the TMT is a neuropsychological test with a “floor effect”, which is the same in the STT. Almost all of the above indexes are not suitable in subjects who could not complete Part B. In our study, in cognitively normal controls, 1.5% subjects could not complete the STT-B. In patients with aMCI and mild AD, even more subjects failed in part B (4.8% and 30.4%). Therefore, we developed an index named “STT-1 min”, which means the number that participants correctly connect within the first one minute. Like the time for completion of the STT-A and STT-B, the STT-B-1 min index could distinguish well among AD, aMCI and controls. The “STT-1 min” is similar with the 25 point scoring method, but saves more time. As we know, both the original TMT and STT are time-consuming. In some AD patients, even more than 10 minutes is needed to complete part B. Adopting the “1 min” index as the score could save time for the test performance and improve efficiency at the same time.

In a sub-sample that completed both the STT and CTT, the STT-A was less than the CTT-A while the STT-B was greater than the CTT-B, which indicated that the interference burden in shape in the STT was heavier than that of color in the CTT. Meanwhile, our cognitively normal subjects spent more time to complete the STT-A and B compared with Americans or Japanese in the TMT-A and B 3,23]. The results suggest that the current STT is more difficult than the original TMT, and that the part B of the STT is harder than the CTT as well. This must be taken into consideration when comparing our data to western countries.

This study evaluated a new variant form of the TMT that fits the cultural background of the Chinese population. With a relatively large sample size, the performance of the cognitively normal older population on this new test was presented. The diagnostic value for identifying aMCI and AD with the STT was also evaluated. However, our study also had important limitations. Being a culture-fair test, we did not have data outside the Chinese population. Neither did we include younger subjects which made the data less standardized. In the future, an equivalence study of the TMT, CTT and STT among well-educated, bilingual Chinese population may have great implications for psychometric test development and clinical cultural neuropsychology.

In summary, the TMT is a sensitive test of visual search and sequencing. The STT is a meaningful attempt to create a “culture-fair” version of the TMT in addition to the CTT. Further validation studies are needed to extensively evaluate this test.
